# Outcome of inflammatory breast cancer in Moroccan patients: clinical, molecular and pathological characteristics of 219 cases from the National Oncology Institute (INO)

**DOI:** 10.1186/s12885-018-4634-9

**Published:** 2018-07-05

**Authors:** Meriem Slaoui, Abdou Azaque Zoure, Fatima Zahra Mouh, Youssef Bensouda, Mohammed El Mzibri, Youssef Bakri, Mariam Amrani

**Affiliations:** 1 Equipe de recherche ONCOGYMA, Faculty of Medicine and Pharmacy of Rabat, University Mohamed V Rabat, Avenue Mohammed Belarbi El Alaoui – Souissi – BP, 6203 Rabat, Morocco; 2 Unité de Biologie et Recherche Médicale, Centre National de l’Energie, des Sciences et des Techniques Nucléaires, Rabat, Morocco; 3 Pietro Annigoni Biomolecular Research Center (CERBA)/LABIOGENE, University of Ouaga 1 Joseph KI ZERBO, UFR/SVT, Ouagadougou, Burkina Faso; 4 Laboratory of Biochemistry and Immunology, Faculty of Sciences, University of Mohammed V-Rabat, Rabat, Morocco; 5 Institute of Health Sciences Research, (IRSS)/ Department of Biomedical and Public Health, Ouagadougou, Burkina Faso; 6 Faculty of Medicine and Pharmacy of Rabat, University Mohamed V Rabat, Avenue Mohammed Belarbi El Alaoui – Souissi – BP, 6203 Rabat, Morocco; 7 Biochemistry-Immunology Laboratory, Faculty of Sciences Rabat, University Mohammed V – Agdal, Rabat, Morocco

**Keywords:** Inflammatory breast cancer, Molecular subtypes, Morocco, Overall survival, Event-free survival

## Abstract

**Background:**

Usually misdiagnosed, Inflammatory Breast Cancer (IBC) is the most aggressive form of non-metastatic breast cancer. This orphan disease is more frequent in North Africa. Despite intensive treatment, the survival rate remains very low.

**Methods:**

We have retrospectively studied all breast cancer cases diagnosed at the National Oncology Institute (INO), Rabat between 2005 and 2010. We have collected 219 cases of women with metastatic and non-metastatic IBC. Data have been obtained from patients’ personal medical files over a follow-up period of 5 years. We have described IBC’s clinicopathological features and analyzed its clinical outcome using SPSS software. HR (hazard Ratio) was calculated using Cox regression analysis.

**Results:**

The frequency of IBC cases is 4.05%. The majority of our patients (65.3%) were under 50 years old. The most prevalent molecular subtype was Luminal A (38.7%) followed by Luminal B HER2+ (27.9%) and Triple negative (21.6%).

During the follow-up period, 72 patients (32.9%) had recurrence and 40 patients (18.3%) died. The 3-year OS (Overall Survival) and EFS (Event Free Survival) of non-metastatic patients were 70.4 and 46.5% respectively, while in the metastatic disease, the 3-year OS was only 41.9%. In non-metastatic women, we observed a higher rate of EFS associated to Selective estrogen receptor modulation treatment (*p* = 0.01), and a lower rate EFS in triple negative breast cancer patients (*p* = 0.02). In univariate analysis, we found that EFS rate is lower in patients presenting Triple Negative tumors when compared to other molecular subtypes (HR: 3.54; 95%CI: 1.13–11.05; *p* = 0.02). We also found that Selective estrogen receptor modulation treatment is associated with higher EFS rate (HR: 0.48; 95%CI: 0.07–0.59; *p* = 0.01).

**Conclusions:**

IBC in Morocco shows similar characteristics to those in North African countries; however, survival rates are still the highest when compared with neighboring countries. Collaborative studies with prospective aspects are warranted to establish the epidemiological profile and understand the high frequencies of IBC in North Africa. More studies on molecular markers are also needed to improve IBC patients’ management and eventually their survival rate.

## Background

Breast cancer is the most prevalent malignancy in women with more than a million and half new cases diagnosed annually [1].

Inflammatory breast cancer (IBC) is however uncommon, and considered as a rare type of breast cancer. Usually misdiagnosed, IBC is the most aggressive form of non-metastatic breast cancer [2]. IBC is characterized by rapid proliferation and several skin changes such as redness, orange skin, edema, ulceration and warmth [3, 4]. The diagnosis of this disease is based on clinical characteristics. Despite all intensive treatments, this study population still shows a very low survival rate [5]. IBC is usually associated with negative hormone receptors especially Estrogen receptor, positive Human Epidermal Growth Factor Receptor-2 (HER2), advanced stages and more metastasis [6].

IBC is more frequent in North Africa with 5% in Morocco, 6% in Tunisia and 11% in Egypt, while in America, only 2.5% of breast cancers are classified as IBC [6–9]. These striking differences in IBC frequencies around the world are still misunderstood. In spite of all the scientific advances in medical research tackling this disease, the identification of risk factors directly related to IBC is inconclusive. Studies suggest that infectious agents, primarily Mouse Mammary Tumor Virus, represent the most probable etiology [10, 11]. Various studies have reported the suspicion of risk factors such as exposure to exogenous hormones, high fat intake, ethnicity, young age, heredity and socio-economic level [12–15]. Still, none of these etiological factors have been proven to be directly linked to IBC.

IBC is still under-studied in Morocco, and to our knowledge, only one published study on this special breast cancer entity is counted [7]. For this reason, we have conducted this relatively large retrospective study of inflammatory breast cancer patients diagnosed at the National Oncology Institute (INO) in Rabat. This study aims at describing clinicopathological features, molecular characteristics and risk factors in a set of Moroccan inflammatory breast cancer patients over a period of 5 years and at analyzing prognostic factors and survival.

## Methods

### Study design and population

Our study population consists of Moroccan women diagnosed with breast cancer and/or followed up at the National Oncology Institute in Rabat, Morocco from January 2005 until December 2010. A total of 5400 breast cancer patients has been recorded. Medical files have been reviewed, and confirmed inflammatory breast cancer cases have been selected for the purpose of this study. At the end, we have collected 219 cases of women diagnosed with metastatic and non-metastatic inflammatory breast cancer.

Inclusion criteria: all Moroccan women diagnosed with IBC during the study period at the National Oncology Institute. We have excluded patients with incomplete medical files and patients without histological confirmation of breast cancer.

Patients’ ages ranged between 26 to 75 years. The mean age of women at diagnosis was 47 ± 10.3.

### Data collection

Data has been obtained from patients’ personal medical files. The medical records have then been retrospectively reviewed and collected using SPSS-software 13.0. For each case, we have collected all information on age, parity, body mass index, hormonal status, familial history of breast cancer, clinical as well as pathological data, and follow-up.

Histological type has been updated according to the WHO classification of breast tumors of 2012 (World Health Organization) [16]. Tumor pTNM (pathological Tumor Node Metastasis) staging is consistent with the seventh edition of AJCC classification (American Joint Committee on Cancer) of 2009. Tumor grade has been assessed according to Scarff-Bloom & Richardson (SBR) grading system, amended by Ellis and Elston [17].

Estrogen and Progesterone receptors (ER and PR) were considered positive when at least 10% of the tumor cells showed nuclear expression.

Immuno-histo-chemical expression of Her 2 has been defined based on cytoplasmic membrane staining of the infiltrative component according to the American Society of Clinical Oncology (ASCO) [18]. Fluorescent in situ hybridization (FISH) has been performed to assess Her 2 amplification in 2+ borderline cases.

According to ER, PR and Her2 status, breast cancer cases have been classified into five subgroups: Luminal A (ER+/PR+/Her2-), Luminal B Her2- (ER+/PR- or lower than 20% /Her2-), Luminal B Her+ (ER+/PR+ or - /Her2+), Her2 (ER-/PR-/Her2+) and triple negative (ER-/PR-/Her2-) [19].

Treatment data such as: surgery type (total mastectomy/Partial mastectomy), chemotherapy, radiotherapy, targeted therapy and hormone therapy have been collected from patients’ medical files. During the study period, selective estrogen receptor modulators (SERM) were being used as hormone therapy.

### Follow-up

Patients were followed up until December 2012. Event free survival (EFS) was calculated from the date of neoadjuvant chemotherapy to the date of loco-regional recurrence or distant metastasis. Overall survival (OS) was calculated from the date of histological diagnosis to the date of death. The follow-up was carried out by checking the status of patients in their personal medical files.

### Statistical analysis

Statistical analysis has been assessed by SPSS 13.0 software (IBM), while descriptive variables have been expressed as means ± SD. Calculation of survival rates has been performed by the Kaplan-Meier method and compared using the Log-rank test. Patients lost to follow-up were considered as a censored event.

Hazard ratios have been calculated using Cox regression analysis and assumptions of Cox proportional hazards regression were checked graphically using “log-log” plots.

## Results

### Clinical and pathological data

The mean age in our series was 47 ± 10.3 years with extreme ages of 26 years and 75 years. Clinical and pathological results are listed in Tables 1 and 2. The majority of our patients (65.3%) were aged under 50 years. Only 31.4% were nulliparous and almost half of the patients had more than three full-term pregnancies. Pre-menopausal women were as many as post-menopausal women; only 35.3% of patients had normal body mass index, while 63.5% were overweight or obese.

**Table 1:** Clinical data in all inflammatory breast patients

Variables	Number of patients	percentage (%)
Age		
˂30y	13	5.9
31–40	46	21.0
41–50	84	38.4
˃50y	76	34.7
Nulliparity		
Yes	66	31.4
No	144	68.6
Unknown	9	–
Number of full-term pregnancies		
0	66	31.5
1–2	43	20.6
3–4	48	23.0
≥ 5	52	24.9
Unknown	10	–
Menopausal staus		
Pre-menopausal	113	51.6
Post-menopausal	106	48.4
Familial history of BC		
Yes	28	26.2
No	79	73.8
Unknown	112	–
BMI		
Underweight	2	1.2
Normal	60	35.3
Overweight	50	29.4
Obese	58	34.1
Unknown	49	–
Peau d’orange		
Yes	155	70.8
No	64	29.2
Oedema		
Yes	45	20.5
No	174	79.5
Redness		
Yes	100	54.3
No	119	45.7
Palpable mass		
Yes	55	25.1
No	164	74.9
Side		
Right breast	102	46.6
Left breast	115	52.5
Bilateral	2	0.9
Metastatic disease		
Yes	66	30.1
No	153	69.9

**Table 2:** pathological data in inflammatory breast cancer tumors

Variables	Number of patients	Percentage (%)
ER		
Positive	99	55.6
Negative	79	44.4
Unknown	41	–
PgR		
Positive	124	69.7
Negative	54	30.3
Unknown	41	–
Her2		
Positive	41	35.3
Negative	75	64.7
Unknown	103	–
Molecular subtype		
Luminal A	43	38.7
Luminal B Her2 –	5	4.5
Luminal B Her2 +	31	27.9
Her2	8	7.2
Triple negative	24	21.6
Unknown	108	–
Tumor size		
≤ 20 mm	27	15.7
21–50 mm	55	32.0
> 50 mm	90	52.3
Unknown	47	–
Lymph nodes		
N0	66	30.1
N1	84	38.4
N2	45	20.5
N3	24	11.0
Histological type		
Invasive carcinoma of NST	212	96.8
Invasive lobular carcinoma	4	1.8
Others	3	1.4
Vascular invasion		
Yes	119	54.3
No	100	45.7
SBR grade		
SBR I	15	7.1
SBR II	110	52.1
SBR III	86	40.8
Unknown	8	–

At the time of diagnosis, sixty-six women had metastatic disease (30.1%). The most prevalent molecular subtype was Luminal A (38.7%) followed by Luminal B HER2+ (27.9%) and Triple negative (21.6%).

Mean tumor size was 6.27 cm, and the majority of patients (52.3%) had tumors sized more than 5 cm. Vascular invasion was found in 119 patients (54.3%). High SBR (SBR II and SBR III) grades were observed in 92.9% of the tumors, and most of patients had invaded axillary lymph nodes (69.9%).

### Treatment

Neoadjuvant chemotherapy was administered to 95.4% of patients: 70.3% received Anthracyclines-based chemotherapy, 23.9% received Anthracyclines and taxanes regimen and only 5.7% took taxanes only. 125 women (57.1%) underwent radical surgery. Adjuvant chemotherapy and Herceptine were administered respectively in 22.8 and 17.4% of the cases. After surgery, 47.5% of the patients received radiotherapy while only 28.3% received SERM (Table 3).

**Table 3:** Treatment data for IBC cases

Treatment	Number of patients	Percentage (%)
Neoadjuvant Chemotherapy		
Yes	209	95.4
No	10	4.6
Mastectomy		
Yes	125	57.1
No	94	42.9
Adjuvant Chemotherapy		
Yes	50	22.8
No	169	77.2
Herceptine		
Yes	38	17.4
No	181	82.6
Radiotherapy		
Yes	104	47.5
No	115	52.5
SERM treatment		
Yes	62	28.3
No	157	71.7

### Survival and outcome

**Fig. 1:**
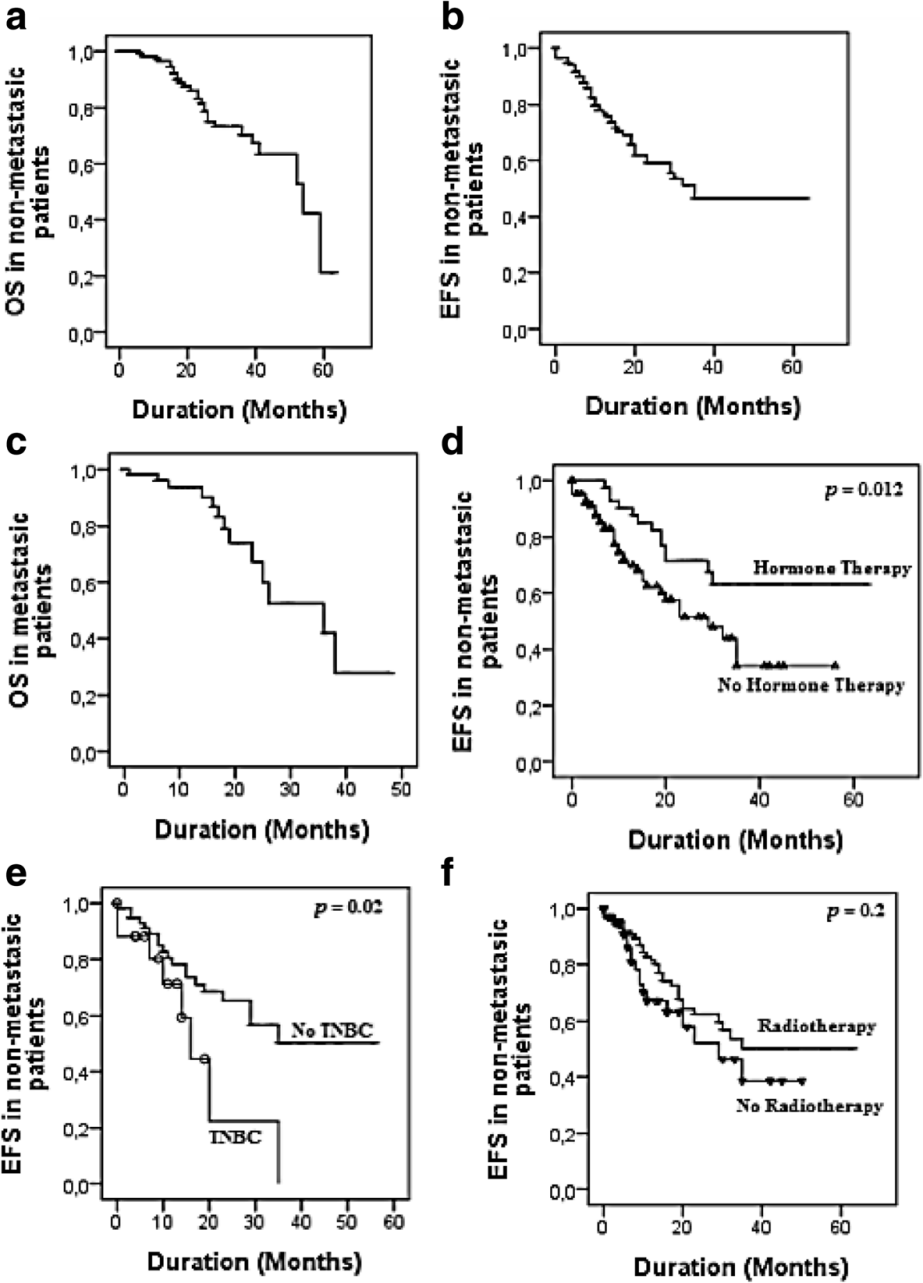
Outcomes (OS and/or EFS) in metastatic and non-metastatic IBC patients (**a, b** and **c**), EFS in TNBC patients (**e**), and impact of Hormone therapy and Radiotherapy (**d** and **f**). (OS: Overall survival; EFS: Event-Free Survival)

Median follow-up was 13 months with a range of 1–63 months. During the follow-up period, 72 patients (32.9%) had recurrence and 40 patients (18.3%) died, while 19 patients (8.67%) were lost to follow-up. The results of Kaplan-Meier analysis are reported in Fig. 1. The 3-year OS and EFS of non-metastatic patients were 70.4 and 46.5% respectively, while in metastatic disease, the 3-year OS was only 41.9% (Fig. 1). In non-metastatic women, we observed a higher EFS rate associated to SERM treatment with a significant difference (*p* = 0.01), and a lower EFS rate in TNBC patients (*p* = 0.02), while the other parameters did not show significant results in Kaplan-Meier analysis.

Univariate and multivariate analysis of EFS and OS are represented in Table 4. In univariate analysis, we found that EFS rate is lower in patients presenting left breast tumors or bilateral tumors (HR: 1.92; 95%CI: 1.07–3.44; *p* = 0.02 - HR: 10.32; 95%CI: 1.32–80.47; *p* = 0.02), and TNBC tumors when compared to other molecular subtypes (HR: 3.54; 95%CI: 1.13–11.05; *p* = 0.02). We also found that SERM treatment is associated with a higher EFS rate (HR: 0.48; 95%CI: 0.07–0.59; *p* = 0.01). The multivariate model shows that the EFS rate in non-metastatic patients is higher in women aged more than 50 years (HR: 0.06; 95%CI: 0.00–0.61; *p* = 0.01) and in patients treated with SERM (HR: 0.09; 95%CI: 0.01–0.72; *p* = 0.02). Univariate analysis for OS did not demonstrate significant associations and no parameter showed close statistical significance (Table 4).

**Table 4:** Univariate and Multivariate Cox analysis for Overall survival and Event-Free Survival in non-metastatic patients

Parameters	Event Free Survival						Overall Survival		
	Univariate analysis			Multivariate analysis			Univariate analysis		
	HR	95% CI	*p*	HR	95% CI	*p*	HR	95% CI	*p*
Side									
Right breast	1			1			1		
Left breast	1.92	1.07–3.44	**0.02**	2.87	0.98–8.42	0.05	1.36	0.61–3.01	0.44
Bilateral	10.32	1.32–80.47	**0.02**	–	–	–	5.14	0.65–40.65	0.12
Obesity^a^									
No	1			1			1		
Yes	1.36	0.72–2.54	0.33	1.45	0.55–3.85	0.44	1.80	0.76–4.27	0.17
SBR Grade									
I	1						1		
II	0.63	0.18–2.16	0.46				0.67	0.14–3.09	0.61
III	1.33	0.40–4.38	0.63	–	–	–	1.00	0.22–4.48	0.99
N status									
N-	1						1		
N+	1.55	0.79–3.04	0.19	–	–	–	1.55	0.61–3.91	0.35
Age^a^									
˂30y	1			1			1		
31–40	0.89	0.29–2.67	0.84	0.24	0.03–1.86	0.17	2.60	0.32–20.76	0.36
41–50	0.66	0.22–1.97	0.46	0.20	0.02–1.60	0.12	1.29	0.16–10.42	0.81
˃50y	0.62	0.20–1.95	0.46	0.06	0.00–0.61	**0.01**	2.18	0.25–18.36	0.47
ER									
Negative	1						1		
Positive	0.69	0.38–1.27	0.23	–	–	–	2.05	0.85–4.98	0.11
PgR									
Negative	1						1		
Positive	0.88	0.46–1.66	0.69	–	–	–	1.03	0.42–2.53	0.94
Her2									
Negative	1						1		
Positive	0.77	0.35–1.70	0.53	–	–	–	0.42	0.04–3.81	0.44
Molecular subtype^a^									
Luminal A	1			1			1		
Luminal B Her2 –	2.57	0.68–9.64	0.16	0.86	0.15–4.82	0.86	2.57	0.78–2.60	0.98
Luminal B Her2 +	1.78	0.59–5.34	0.29	0.31	0.07–1.34	0.11	0.43	0.04–4.23	0.47
Her2	0.57	0.06–4.91	0.61	0.11	0.01–1.18	0.06	0.57	0.05–3.81	0.98
Triple negative	3.54	1.13–11.05	**0.02**	1.72	0.47–6.31	0.41	1.99	1.10–10.00	0.96
Surgery^a^									
No	1			1			1		
Yes	0.73	0.39–1.39	0.34	1.13	0.37–3.49	0.82	0.62	0.23–1.70	0.36
Radiotherapy^a^									
No	1			1			1		
Yes	1.20	0.9–1.6	0.21	0.88	0.28–2.73	0.82	1.23	0.81–1.87	0.32
SERM treatment									
No	1			1			1		
Yes	0.48	0.07–0.59	**0.01**	0.09	0.01–0.72	**0.02**	1.01	0.69–1.49	0.92

^a^: variables being adjusted for the multivariate model; significant *p* values are in boldface

## Discussion

In this study, we have intended to investigate IBC’s clinical, molecular and pathological features, and analyze survival in Moroccan patients diagnosed with IBC between 2005 and 2010.

IBC is more frequent in North African countries, especially in Tunisia and Egypt where frequencies are 5 and 6% respectively. In our series, the frequency of IBC cases was 4.05%, which agrees with a previous study conducted at the same institute where authors have found an occurrence of 5% of all breast cancer cases [7].

A number of important epidemiological studies have found that IBC occurs at a younger age than non-inflammatory breast cancer [10]. Indeed, 65.3% of our IBC patients were younger than 50 years, while in Algeria the percentage was 59.8%. On the other hand, the National Cancer Institute’s Surveillance, Epidemiology, and End Results (SEER) program has shown that only 34.7% of IBC patients were aged less than 50 years [20]. We have also noted some differences in median age between Algerian, Tunisian, Moroccan and American IBC series. Tunisian patients represent the youngest age with a median age of 43.5 years [21], followed by Moroccan and Algerian patients with a median age of 47 years and 48.5 years, respectively [7, 22]. Whereas American patients from the SEER program have shown the higher median age, 56 years [20]. These comparisons show that IBC might occur at younger age in North African populations compared to the American one. We may explain these differences by the possible viral etiology especially Mouse Mammary Tumor Virus Like (MMTV-Like) as described in previous studies led in this area [23, 24].

IBC diagnosis is entirely clinical and well established by AJJC; it is based on the presence of inflammatory signs especially diffuse erythema and oedema of the breast with or without an underlying mass. In the present study, palpable mass was detected in only 25.1% as compared to the Algerian series where it was detected in 31.9% of patients, while in Tunisian patients, the majority of women (76%) had palpable mass at the time of diagnosis [21, 22]. Once again, the Tunisian population shows a different aspect from the Algerian and Moroccan populations.

High BMI is considered as a risk factor for IBC and has been analyzed in several studies but the results are not conclusive [12, 21, 25, 26]. In the Tunisian series, 42% of IBC patients were obese while in our study we have registered a percentage of 34.1%. Data from the Breast Cancer Surveillance Consortium (BCSC) shows that 32.2% of IBC patients had a high BMI [26]. In a French study, we note that IBC patients are less obese, and only 21% of patients presented high BMI [12]. Furthermore, results from a comparative study between North-African series show no significant difference in BMI between IBC and non-IBC patients, but the authors still insist on the need for further studies because of the increasing incidence of obesity among women in North Africa [27].

IBC is known to show pejorative pathological characteristics. Therefore, we have found that 84.3% of the tumors measured more than 2 cm in greatest diameter, which joins the Algerian study findings with 88% of large sized tumors [22]. High SBR grades (SBR II and SBR III) were found in 92.9% of our IBC patients, 80.2% of SEER population [20], 76% of Tunisian patients [21], and 100% of Algerian and Egyptian patients [22, 27]. The comparative study between North African countries (Egypt, Tunisia and Morocco) demonstrate no statistical difference regarding SBR grades [27]. At the molecular level, many studies have documented that IBC is usually correlated to negative hormone receptors and positive HER2 status, which confers to this disease its aggressiveness [2]. The Tunisian study has shown that 52% of IBC tumors were ER-/PR- [28], while in Egypt only 38.9% of the tumors were negative for hormone receptors [27]. The lack of expression of hormone receptors in the Algerian study was 26.7% for ER and 71.8% for PR [22], while in our study IBC tumors were ER- in 44.4% and PR- in 30.3%. According to the comparative study, these disparities between North African countries did not show a significant difference [27].

Studies suggest that about 20~ 40% of IBC cases are triple negative breast cancers [2, 22, 29], which has a worse prognosis and lower survival rates than other breast cancer subtypes. Our study has shown the same range with 21.6% of TNBC tumors, and EFS was also at a lower rate in the TNBC subgroup compared to the other molecular subgroups with a significant difference (*p* = 0.02). The investigation of the seven triple negative subtypes, as described in Lehmann study (*basal-like 1 (BL1), basal-like 2 (BL2), immunomodulatory (IM), mesenchymal (M), mesenchymal stem-like (MSL), luminal androgen receptor (LAR), and unstable (UNS)*), could contribute to resolving the differing clinical behavior when IBC and TNBC coexist [30, 31].

Interestingly and as in the Algerian study [22], the most prevalent subtype in our series was Luminal A followed by luminal B HER2+, unlike the Tunisian study where the most prevalent subtype was TNBC followed by HER2 subtype [32]. Molecular differences between these neighboring countries might be due to environmental and genetic factors that vary from an area to another. Further collaborative studies between these countries are needed.

The role of adjuvant endocrine therapy in the survivorship of IBC patients was clearly investigated in several clinical trials and concluded that SERM treatment is as efficient as chemotherapy in premenopausal breast cancer patients [21, 33]. Our study as well as the Tunisian one shows a significant better EFS in IBC patients who received adjuvant SERM treatment [21].

Contrastingly, the survival rates are higher in our series compared to the Tunisian study. In fact, the 3-year OS and EFS in our series were 70.4 and 46.5% respectively, while in Tunisia rates were 44 and 28%, respectively. This difference is mostly due to the lack of supportive care services and the absence of access to new drugs such as taxanes during the 1990’s, which corresponds to the period of study in Tunisian series [21].

Our study has several strengths. First, the number of patients with IBC is relatively large. Second, the large period that was taken to select participants extended over 6 years. Furthermore, our study represents the first large study including clinical, epidemiological, pathological and molecular characteristics of IBC in Moroccan patients.

This study has also limitations due to its retrospective aspect. Lack of data in some parameters is the major limitation. In addition, the study has been conducted in a single institution. Although it is the reference center of oncology in Morocco, our patients are not representative of the population. We also believe that short median follow-up and loss to follow-up rates could have influenced our survival rates. Finally, socioeconomic conditions have not been investigated, which might have limited access to some drugs like taxanes and Trastuzumab.

## Conclusions

IBC in Morocco shows similar characteristics to those in North African countries; however, survival rates are still the highest when compared with neighboring countries. Collaborative studies with prospective aspects are warranted to establish the epidemiological profile and understand the high frequencies of IBC in North Africa. More studies on molecular markers are also needed to improve IBC patients’ management and eventually their survival rate.

## Abbreviations

AJCC: American Joint Committee on Cancer
BC: Breast cancer
CI: Confidence interval
EFS: Event free survival
ER: Estrogen receptor
FISH: Fluorescent in situ hybridization
Her2: Human epidermal growth factor receptor 2
HR: Hazard ratio
IBC: Inflammatory Breast Cancer
IHC: Immunohistochemistry
LN: Lymph nodes
NST: No Special Type
PgR: Progesterone receptor
pTNM: Pathological Tumor Node Metastasis
SBR: Scarff-Bloom Richardson classification
SERM: Selective estrogen receptor modulation
TNBC: Triple negative breast cancer
WHO: World Health Organization
